# Prevalence of osteoporosis and osteopenia in elderly patients scheduled for total knee arthroplasty

**DOI:** 10.1007/s00402-021-04297-x

**Published:** 2021-12-17

**Authors:** Maximilian M. Delsmann, Constantin Schmidt, Moritz Mühlenfeld, Nico Maximilian Jandl, Christoph Kolja Boese, Frank Timo Beil, Tim Rolvien, Christian Ries

**Affiliations:** 1 Department of Trauma and Orthopaedic Surgery, Division of Orthopaedics, University Medical Center Hamburg-Eppendorf, Martinistraße 52, 20246 Hamburg, Germany; 2 Department of Osteology and Biomechanics, University Medical Center Hamburg-Eppendorf, Lottestraße 59, 22529 Hamburg, Germany

**Keywords:** Osteoporosis, Total knee arthroplasty, Osteoarthritis, Bone mineral density, DXA

## Abstract

**Introduction:**

Osteoporosis is a common comorbidity in elderly patients with osteoarthritis (OA) and may increase perioperative complications in orthopedic surgery (e.g., component migration, periprosthetic fractures). As there is no investigation of bone mineral density (BMD) in elderly patients prior to total knee arthroplasty (TKA) in Europe, we investigated this issue with a particular focus on a potential treatment gap.

**Materials and methods:**

We assessed the BMD by dual-energy X-ray absorptiometry (DXA) in 109 consecutive elderly patients (age ≥ 70 years) scheduled for TKA. In addition to a detailed assessment of osteoporosis and osteopenia, the influence of clinical risk factors and radiological OA severity on BMD was evaluated using group comparisons and linear regression models. In addition, we analyzed differences in BMD between patients scheduled for TKA vs. total hip arthroplasty (THA).

**Results:**

Of the included 109 patients, 19 patients (17.4%) were diagnosed with osteoporosis and 50 (45.9%) with osteopenia. In the osteoporotic patients, a clinically relevant underdiagnosis concomitant with a serious treatment gap was observed in 95.0% of the patients. Body mass index, OA grade, and glucocorticoid use were identified as independent factors associated with BMD. No differences in BMD were found between the patients scheduled for TKA vs. THA.

**Conclusions:**

Considering the high prevalence of osteoporosis and osteopenia in elderly patients, DXA screening should be recommended for patients ≥ 70 years indicated for TKA.

## Introduction

Total knee arthroplasty (TKA) represents a successful surgical procedure for treating patients with end-stage osteoarthritis (OA) of the knee for whom nonoperative treatment has been unsuccessful [1]. Several novel technologies have been recently proposed to optimize the outcome of this procedure [2–4]. Knee OA is a highly prevalent joint disorder with an increasing occurrence within the last century [5, 6] that seems to be attributable not only to aging and obesity, but also to multifactorial influences of the modern environment [5, 7]. Therefore, a closer investigation of this disease and the affected elderly patient population is needed to further decipher the influence of medical conditions and comorbidities on onset and outcome, respectively.

Owing to the considerable risk of osteoporosis in elderly patients with OA [8–10], the International Society for Clinical Densitometry (ISCD) recommends an assessment of bone mineral density (BMD) in women aged ≥ 65 years and men aged ≥ 70 years prior to orthopedic surgery, including TKA, to avoid adverse outcomes [11]. In TKA, poor bone status has been associated with intraoperative and periprosthetic fractures, migration of components, and aseptic loosening [12–16]. Furthermore, decreased BMD in the proximal tibia has been identified as a risk factor for migration of the tibial component [16]. In this context, a circumferential cortical bone support of the proximal tibial component appears mandatory to prevent implant migration. Nonetheless, BMD assessment prior to TKA plays a minor role in a clinical setting so far and is often not performed on a routine basis.

A significant prevalence of osteoporosis in patients undergoing arthroplasty has been reported previously, but methodological differences, such as a broad age range, pre- vs. postoperative evaluation, no differentiation between knee vs. hip replacement were limiting factors, resulting in a heterogeneous osteoporosis frequency ranging from 20.0 to 59.8% [8, 9, 17–20]. In addition, there appears to be a geographic difference in the prevalence of osteoporosis before total joint arthroplasty, with more than twice as high values in Asian than in more western countries [8, 9, 17–19]. Importantly, no study exists investigating the occurrence of osteoporosis in an elderly population prior to TKA in Europe. In this study, we aimed to analyze for the first time BMD measured by dual-energy X-ray absorptiometry (DXA) in a consecutive series of elderly OA patients aged ≥ 70 years (i.e., unbiased approach) in a Central European country before TKA, focusing on a possible treatment gap.

## Methods

### Study cohort

We retrospectively analyzed 109 consecutive patients aged ≥ 70 years who underwent TKA due to end-stage OA in our department in 2018 and 2019. Two patients who initially presented to our department were excluded due to cancer with skeletal metastases (*n* = 1) and inoperability due to severe disease (*n* = 1). Demographic parameters (age, sex, BMI) and relevant clinical risk factors for osteoporosis and increased fracture risk (previous fractures, rheumatoid arthritis, oral glucocorticoid intake for longer than three months in the past or at present, regular consumption of more than three units of alcohol per day, tobacco use, diabetes mellitus) were obtained in all patients. Furthermore, as part of routine clinical workup, BMD was determined preoperatively before TKA by DXA according to the ISCD recommendation [11]. For patients with an indication for osteoporosis treatment, bone-specific drugs were recommended or initiated according to the osteoporosis guidelines of the Dachverband Osteologie (DVO) [21]. The severity of OA was determined from preoperative radiographs using the Kellgren–Lawrence score [22]. To interpret the DXA data from the TKA patients in this study, we compared the results with 268 consecutive elderly patients scheduled for THA who had been analyzed in a previous study [10]. This retrospective study was approved by the local ethics committee (2021-300036-WF) and was performed in accordance with the most recent version of the Declaration of Helsinki.

### Dual-energy X-ray absorptiometry (DXA)

As part of routine clinical workup, we assessed bone mineral density (BMD) at the left and right proximal femur and lumbar spine (L1–L4) in all patients by dual-energy X-ray absorptiometry (DXA; Lunar Prodigy enCore 2007, GE Healthcare; Madison, WI, USA) according to the German osteoporosis guidelines (DVO). All measurements were performed within three months before TKA. *T*-scores expressing BMD standard deviations for young, sex-matched healthy adults were generated using the manufacturer's software. Based on the *T*-score, osteoporosis and osteopenia were diagnosed according to World Health Organization (WHO) guidelines (i.e., normal *T*-score > − 1.0, osteopenia *T*-score > − 2.5 ≤ − 1.0, osteoporosis *T*-score ≤ − 2.5).

### Statistical analysis

GraphPad Prism^®^ (version 9.0, GraphPad Software, La Jolla, CA) and SPSS^®^ statistical program (version 26.0, IBM, Armonk, New York, USA) were used for statistical analyses. Continuous variables are given as mean ± standard deviation (SD) and categorical variables are expressed as number and percentage. The normality of the data distribution was tested by using the Shapiro–Wilk test. Unpaired data from two groups were tested for significance using Student’s *t* test for normally distributed data and Mann–Whitney *U* test for non-normally distributed data. One-way ANOVA with Tukey’s post hoc analysis was performed to analyze differences between more than two normally distributed groups multiple comparisons for data. Kruskal–Wallis test with Dunn’s post hoc test for multiple comparisons was used for non-normally distributed data. Age- and weight-associated changes in *T*-scores were analyzed with linear regression analysis. The *p* value, the coefficient of determination *R*^2^ and the 95% confidence interval (CI) of the respective regression slopes were determined. To determine the independent influence of gender, age, BMI, or Kellgren–Lawrence score on the *T*-score, a multiple linear regression model was performed using the “enter” method to evaluate the influence of all variables simultaneously. Statistical significance was set to a 2-tailed *p* value of 0.05.

## Results

**Fig. 1:**
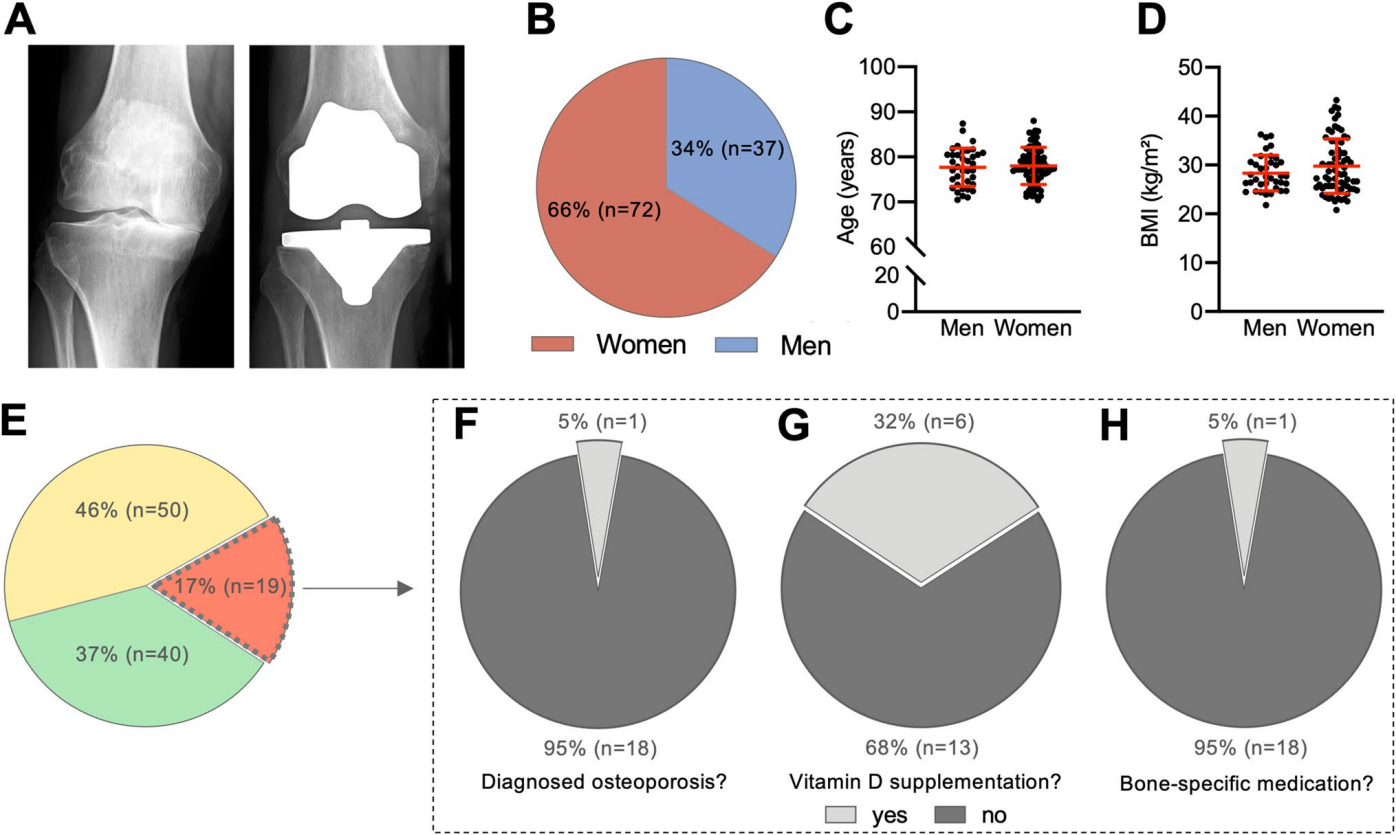
Demographic characteristics and DXA results in patients scheduled for TKA. **A** Radiographs of a representative case of end-stage medial osteoarthritis before (left panel) and after (right panel) TKA. **B** Sex distribution in the patient cohort. **C** Age and **D** BMI distribution between the sexes. **E** DXA results divided into the proportion of patients with osteoporosis (red), osteopenia (yellow), and normal BMD (green). **F** Evaluation of osteoporosis patients concerning the status of prior diagnosis establishment, **G** vitamin D substitution, and **H** specific antiresorptive treatment

We examined 109 consecutive patients (72 women and 37 men) with end-stage OA awaiting TKA (Fig. 1A, B). There was no difference between the sexes in terms of age (Fig. 1C) and BMI (Fig. 1D). Clinical risk factors for osteoporosis did not differ between women and men (Table 1). Of the total patient cohort, 12/109 patients (11.0%) suffered at least one fracture in their history, with vertebral fractures in seven (6.4%) and peripheral fractures in five patients (4.6%). Eight patients (7.3%) had a confirmed diagnosis of rheumatoid arthritis and 20 patients (18.3%) of type 2 diabetes mellitus. In addition, eight patients (7.3%) reported current or past use of oral glucocorticoids for more than three months, while seven patients (6.4%) reported high-risk alcohol use, and twelve patients (11.0%) were smokers. All demographic data as well as risk factors are summarized in Table 1, subdivided into women and men.

**Table 1:** Demographic data, medical history, risk factors, and DXA results in male and female patients scheduled for TKA

	Women *n* = *72*	Men *n* = *37*	*p*
*Patient characteristics*			
Age (years)	78.0 ± 4.1	77.7 ± 4.3	0.705
Height (cm)	163.6 ± 7.1	176.1 ± 6.2	**< 0.0001**
Weight (kg)	79.3 ± 14.0	87.9 ± 12.3	**0.002**
BMI (kg/m^2^)	29.8 ± 5.6	28.3 ± 3.7	0.163
Kellgren–Lawrence score	3.5 ± 0.6	3.7 ± 0.6	0.064
*Medical history and risk factors*			
Previous fragility fractures	9/72 (12.5%)	3/37 (8.1%)	0.488^#^
Vertebral fractures	4/72 (5.6%)	3/37 (8.1%)	0.607^#^
Peripheral fractures	5/72 (7.0%)	0/37 (0.0%)	N/A
Rheumatoid arthritis	7/72 (9.7%)	1/37 (2.7%)	0.183^#^
Diabetes mellitus type 2	11/72 (15.3%)	9/37 (24.3%)	0.248^#^
Glucocorticoids	7/72 (9.7%)	1/37 (2.7%)	0.183^#^
Three or more units alcohol/day	4/72 (5.6%)	3/37 (8.1%)	0.607^#^
Current smoking	6/72 (8.3%)	6/37 16.2%)	0.213^#^
*DXA results*			
*T*-Score_Min_	− 1.3 ± 1.2	− 1.1 ± 1.4	0.406
*Z*-Score_Min_	0.0 ± 1.1	− 0.4 ± 1.4	0.911
BMD_Min_	0.8 ± 0.2	1.0 ± 0.2	**0.003**
Osteoporosis	13/72 (18.1%)	6/37 (16.2%)	0.810^#^
Osteopenia	37/72 (51.4%)	13/37 (35.1%)	0.107^#^
Normal BMD	22/72 (30.6%)	18/37 (48.7%)	0.063^#^

*n* number of patients, *T-score*_*Min*_ minimum *T*-score, *Z-score*_*Min*_ minimum *Z*-score, *BMD*_*Min*_ minimum bone mineral density, *BMD* bone mineral density

^#^Determined by the *χ*^2^ test. The results are presented as mean ± SD or *n* (%). Bold indicates significant differences (*p* < 0.05)

The analysis of DXA results showed that only 40 patients (36.7%) awaiting TKA had normal *T*-scores, whereas the majority (*n* = 69; 63.3%) had reduced BMD (Fig. 1E). Specifically, osteopenia was detected in 50 (45.9%) and osteoporosis in 19 patients (17.4%). While osteoporosis had been previously diagnosed in only 1/19 patients, it was first diagnosed in the remaining 18 patients (Fig. 1F), revealing a clinically relevant underdiagnosis in 95.0% of the cases. As a result, only six patients (31.6%) received vitamin D supplementation (Fig. 1G). Furthermore, only the one patient (5.3%) with previously diagnosed osteoporosis received a bone-specific medication (i.e., antiresorptive treatment) (Fig. 1H). When comparing the DXA results between women and men, the minimum *T*- and the *Z*-score did not differ. However, as expected, the minimum absolute BMD value was significantly higher in men than in women (Table 1; *p* = 0.003).

**Fig. 2:**
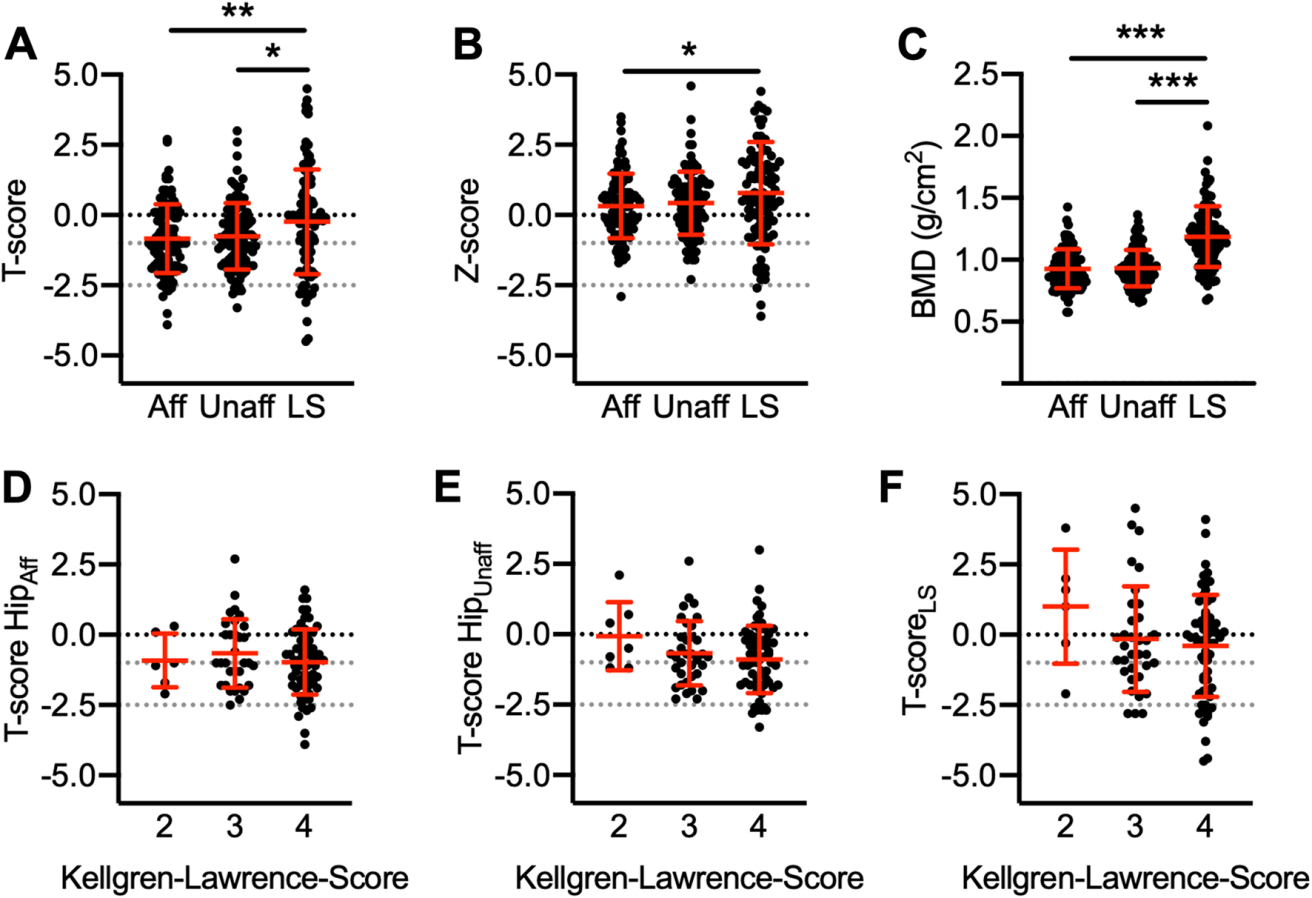
Comparison of DXA results between different measurement sites and radiological OA-grades. **A** DXA *T*-scores of both hips (affected and unaffected leg) and the lumbar spine, **B** *Z*-scores, and **C** BMD. **D** Comparison of the hip *T*-scores of the affected leg, **E** unaffected leg, **F** and lumbar spine between individual Kellgren–Lawrence scores of the operated knee. Ordinary one-way ANOVA and Tukey’s multiple comparison test was used in all panels. *Aff* affected leg, *Unaff* unaffected leg, *LS* lumbar spine, *T-score Hip*_*Aff*_ Hip *T*-score of the affected leg, *T-score Hip*_*Unaff*_ Hip *T*-score of the unaffected leg, *T-score*_*LS*_ lumbar spine *T*-score. *p < 0.05, **p < 0.01, ***p < 0.0001

Analyzing the different DXA measurement sites, the hip *T*-score was significantly lower in both the affected (i.e., scheduled for TKA) (− 0.8 ± 1.2; *p* = 0.009) and unaffected leg (i.e., contralateral side) (− 0.8 ± 1.1; *p* = 0.028) as compared to the lumbar spine (− 0.2 ± 1.9; Fig. 2A), while the *Z*-score differed significantly only between the hip in the affected leg (0.3 ± 1.1 vs. 0.8 ± 1.8; *p* = 0.048) and lumbar spine (Fig. 2B). The BMD of the hips was also significantly lower in the affected (0.9 ± 0.2; *p* < 0.0001) and unaffected leg (0.9 ± 0.1; *p* < 0.0001) as compared to the lumbar spine (1.2 ± 0.2; Fig. 2C). No significant differences in BMD were evident in relation to the OA severity levels as measured by the Kellgren–Lawrence score (Fig. 2D–F).

**Fig. 3:**
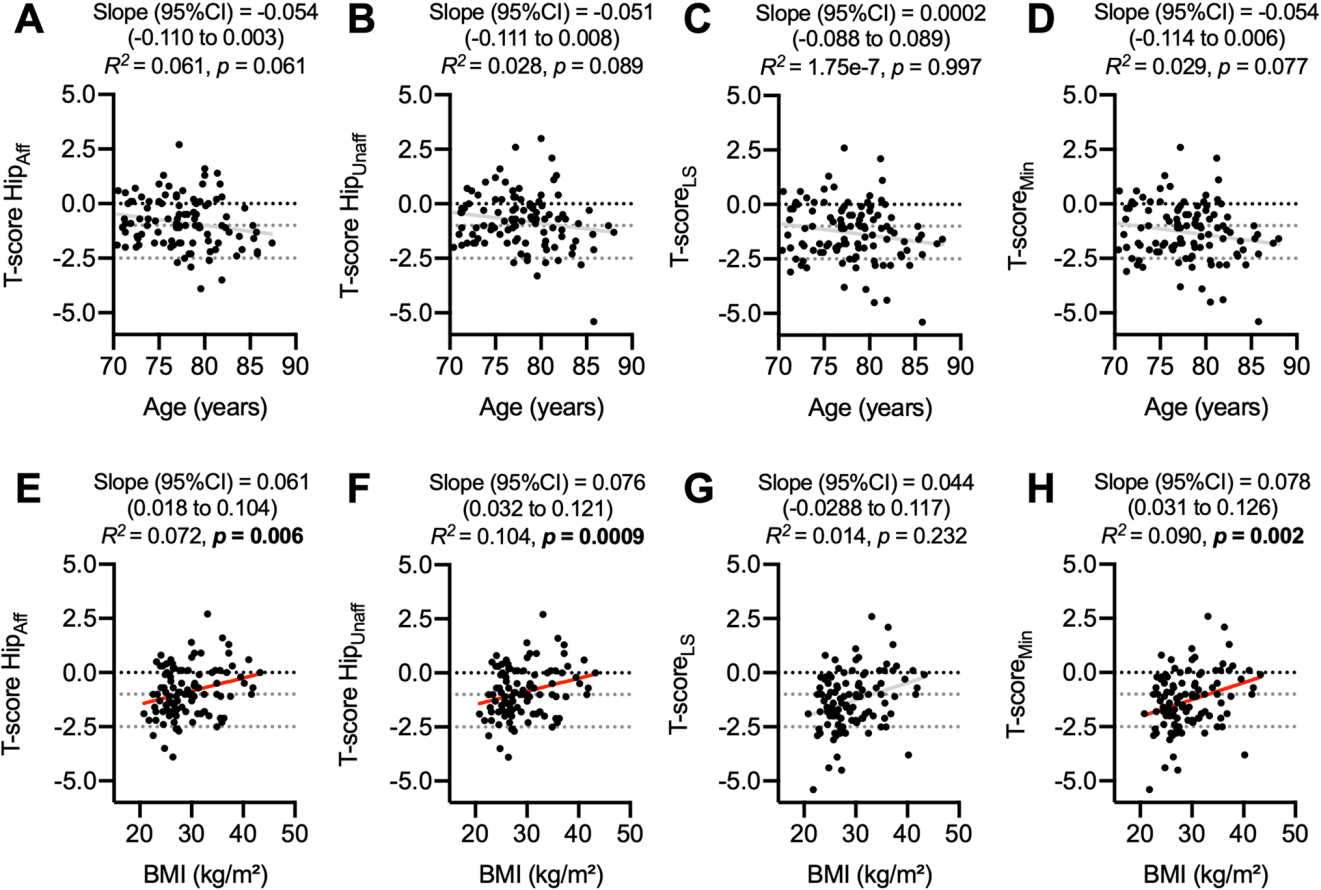
Associations between age or BMI and DXA *T*-score. **A** Linear regression analysis of hip *T*-scores of the affected leg, **B** unaffected leg, **C** lumbar spine, **D** and minimum *T*-score as a function of age (**E**–**H**, respective) as well as BMI, respectively. Linear regression models were calculated in all panels. *T-score Hip*_*Aff*_ Hip *T*-score of the affected leg, *T-score Hip*_*Unaff*_ Hip *T*-score of the unaffected leg, *T-score*_*LS*_ lumbar spine *T*-score, *T-score*_*Min*_ minimum *T*-score, *BMI* body mass index

Interestingly, there was no association between age and *T*-score at any measurement site in our study cohort (Fig. 3A–D). However, there was a positive linear association between BMI and *T*-scores in the affected (Fig. 3E) and unaffected leg (Fig. 3F). Although there was no association with lumbar spine measurements (Fig. 3G), minimum *T*-score also significantly increased with higher BMI (Fig. 3H).

Using a multiple linear regression model, we identified the Kellgren–Lawrence score, BMI, and glucocorticoid use as independent factors associated with the *T*-score (Table 2). Specifically, a higher Kellgren–Lawrence score was associated with a lower minimal *T*-score, while a lower BMI represented an independent influencing factor for a lower minimal *T*-score and a lower hip *T*-score of the affected side. The use of glucocorticoids was additionally associated with a lower *T*-score at the lumbar spine.

**Table 2:** Multiple linear regression model analyzing the independent factors associated with BMD *T*-scores

	*T*-Score_Min_			*T*-Score Hip_Aff_			*T*-Score_LS_		
	*ß*	*T*	*p*	*ß*	*T*	*p*	*ß*	*T*	*p*
(Intercept)		0.753	0.453		0.587	0.558		− 0.049	0.961
Age	− 0.124	− 1.411	0.161	− 0.143	− 1.529	0.130	0.030	0.302	0.763
KLS	− 0.247	− 2.779	**0.006**	− 0.079	− 0.826	0.411	− 0.161	− 1.632	0.106
Sex	− 0.100	− 1.102	0.273	− 0.020	− 0.205	0.838	− 0.159	− 1.590	0.115
BMI	0.263	2.955	**0.004**	0.233	2.464	**0.016**	0.112	1.127	0.262
Corticosteroids	− 0.227	− 2.569	**0.012**	− 0.276	− 2.938	**0.004**	− 0.214	− 2.188	**0.031**
*R*^2^ adjusted	0.190**			0.140**			0.067**		

*T-score*_*Min*_ minimum *T*-score, *T-score Hip*_*Aff*_ Hip *T*-score of the affected leg, *T-score*_*LS*_ lumbar spine *T*-score, *KLS* Kellgren–Lawrence score, *BMI* body mass index

Bold indicates significant independent predictors (*p* < 0.05). **p < 0.001 for the multiple linear regression model. Regression coefficients were estimated for sex (0 = male, 1 = female) and glucocorticoid use as a bivariate variable (0 = no, 1 = yes). Age and BMI were given in years (year) and kg/m^2^, respectively

**Fig. 4:**
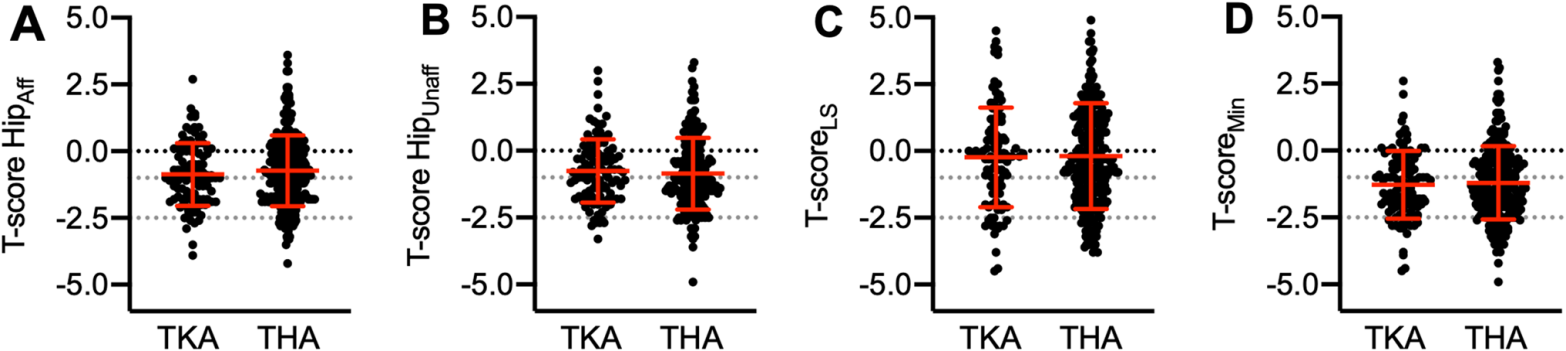
Differences between *T*-scores in elderly TKA vs. THA patients. Different measurement sites are displayed. Comparison of hip *T*-scores of **A** the affected leg, **B** unaffected leg, **C** lumbar spine, **D** and minimum *T*-score. *t* test was used in all panels. *T-score Hip*_*Aff*_ Hip *T*-score of the affected leg, *T-score Hip*_*Unaff*_ Hip *T*-score of the unaffected leg, *T-score*_*LS*_ lumbar spine *T*-score, *T-score*_*Min*_ minimum *T*-score

Finally, we compared the *T*-scores of patients scheduled for TKA with a previously published cohort of 268 THA patients (178 women and 90 men) [10]. The two cohorts were similar in age (TKA: 77.9 ± 4.1 years vs. THA: 78.2 ± 4.8 years; *p* = 0.48), but the TKA patients presented with significantly higher BMI values (TKA: 29.3 ± 5.0 kg/m^2^ vs. THA: 27.5 ± 4.1 kg/m^2^; *p* = 0.0003). There were no significant differences between the two groups in the hip *T*-scores of the affected (Fig. 4A) or unaffected leg (Fig. 4B), lumbar spine (Fig. 4C), or the minimum *T*-score (Fig. 4D), suggesting that knee and hip OA likely do not affect osteoporosis differently.

## Discussion

In the present study, we aimed to investigate the prevalence of reduced BMD, including potential influencing factors, in consecutive elderly knee OA patients prior to TKA. This study is the first to address this question in a European patient population without preselection of patients with certain risk factors (i.e., unbiased approach). Special focus was also given to the extent to which patients diagnosed with osteoporosis receive adequate treatment.

We demonstrated that a substantial proportion of knee OA patients had osteopenia and osteoporosis in terms of BMD *T*-scores ≤ − 1.0 and ≤ − 2.5, respectively. Through our approach, most were first diagnosed with previously unknown and untreated osteoporosis, indicating a clinically relevant underdiagnosis and treatment gap. Further investigation revealed that especially patients with a low BMI, oral glucocorticoid use, and high Kellgren–Lawrence score were at particular risk for reduced BMD. DXA results did not show differences between the hips of the affected and the unaffected side. Therefore, pain-related unloading of the affected leg seems not to lead to a side-specific difference in BMD of the unilateral hip. Only BMD values in the lumbar spine were significantly higher compared with the hips, probably due to accompanying degenerative changes in the spine [23, 24].

Interestingly, no differences in BMD were observed between elderly patients awaiting TKA and those scheduled for THA. While in our previous study [10], analyzing patients prior to THA, osteoporosis was diagnosed in 18% and osteopenia in 41%, in the present study osteoporosis was diagnosed in 17% and osteopenia in 46%. Also, no significant differences were measured at the individual DXA measurement sites between these two patient cohorts, indicating that the manifestation site of OA is independent of BMD or, in other words, that knee and hip OA equally affect BMD.

There seem to be substantial geographic differences in the occurrence of osteoporosis. In this regard, Asian studies in particular show high osteoporosis rates by DXA in patients undergoing TKA, but the underlying reason is unclear. Specifically, osteoporosis was detected in 59.8% of Chinese postmenopausal women (69.7 ± 8.5 years) prior to TKA [19]. The prevalence is markedly lower in our cohort (18.1%) even though our patients are comparably older (77.9 ± 4.1 years). An analysis performed in Korea also showed a substantially higher prevalence of osteoporosis (50.0% of the total cohort) [17]. A comparison with the prevalence from other studies appears inaccurate, as a solely postoperative assessment of BMD or the lack of distinction between THA and TKA had been considered as confounding factors [8, 9, 18, 25–27]. The data of the present study, which appear to reflect plausible results considering previous studies analyzing prevalence of osteoporosis in the general population [28, 29], represent the first representative and consecutive data analysis on osteoporosis rates in elderly patients awaiting TKA in a Central European country.

Despite the lower prevalence as compared to Asian countries, the clinically relevant underdiagnosis of osteoporosis presents a serious issue, which is inevitably linked to a treatment gap and strengthens the need for routinely performed DXA measurements before TKA. This is an important necessity since patients with osteoporosis present an increased risk of atraumatic or low-energy fractures anyway [30, 31], but also exhibit a special additional risk profile in the context of TKA, namely intraoperative and periprosthetic fractures, component migration, aseptic loosening [12–16, 32, 33].

Especially, considering the increasing trend of the prevalence of osteoporosis [34], the importance for standardized implementation of DXA measurements prior to TKA in elderly patients will increase in the future. Failure of treatment in osteoporotic patients is also an important concern, as appropriate specific osteoporosis therapy can lead to a significantly improved outcome [32, 33, 35–38]. In this way, antiresorptive and osteoanabolic drugs, like bisphosphonates, denosumab or teriparatide, can significantly prevent periprosthetic bone loss after TKA in the tibia and femur, thus effectively reducing the risk of periprosthetic fractures [32, 33, 35, 36, 39]. Periprosthetic bone loss, which can occur regularly and already in the first months after surgery [40, 41], may be a particular relevant problem in osteoporotic patients in terms of skeletal-related complications, and this bone loss can be effectively counteracted by specific osteoporosis therapy, especially in the metaphyseal region [38]. As a result, antiresorptive treatment in patients suffering from osteoporosis after total joint arthroplasty of the lower extremity is associated with a significantly lower revision rate and an almost twofold increase in implant survival [37]. Considering the high prevalence of osteoporosis in elderly patients with potential implications for treatment, the recommendation to routinely perform DXA examination in patients ≥ 70 years of age is strongly highlighted to avoid complications and to ensure long implant survival.

## Conclusion

In elderly patients ≥ 70 years in Central Europe with end-stage OA scheduled for TKA, a substantial proportion was diagnosed with reduced BMD. Most osteoporosis patients did not receive adequate treatment, indicating clinically relevant underdiagnosis and undertreatment. Low BMI, high OA severity, and the use of glucocorticoids were identified as independent factors associated with poor BMD. No differences in BMD were found when comparing patients before TKA and THA. Considering the impact on outcome, general DXA screening for elderly patients ≥ 70 years of age should be recommended before TKA. Population differences need to be respected concerning the prevalence of osteoporosis in orthopedic cohorts.
